# Dangerous Fashionable Folly—Green Artificial Flowers

**Published:** 1862-09

**Authors:** 


### DANGEROUS FASHIONABLE FOLLY—GREEN ARTIFICIAL FLOWERS.

An analysis of green artificial flowers has been made by Prof. Hoffmann, for the Ladies' Sanitary Association; he says:—

"In a dozen of the leaves sent me, analysis has pointed out on an average, the presence of ten grains of white arsenic. I learn from some lady friends that a ball-wreath usually contains about fifty of these leaves. Thus a lady wears in her hair more than forty grains of white arsenic, a quantity which, if taken in appropriate doses, would be sufficient to poison twenty persons. This is no exaggeration, for the leaves sent to me were—some of them at least—only partly colored, others only variegated. In consequence of some inquiries, I have been led lately to pay more than usual attention to the head-dresses of ladies, and I observe that the green leaves are often much larger and more deeply colored than those which I have received.

"Ladies cannot, I think, have the remotest idea of the presence of arsenic in their ornaments. If aware of their true nature, they would be satisfied with less brilliant colors, and reject, I have no doubt, these showy green articles, which have not even the merit of being, as far as coloring is concerned, a truthful imitation of nature."—*Medical News and Library.*

